# The first report of Bathynellacea in the subterranean water of Mongolia: A new species of *Altainella* Camacho, 2020 (Crustacea, Bathynellidae)

**DOI:** 10.3897/BDJ.12.e130024

**Published:** 2024-08-07

**Authors:** Su-Jung Ji, Ana Isabel Camacho, Chi-Woo Lee, Gi-Sik Min

**Affiliations:** 1 Department of Biological Sciences and Bioengineering, Inha University, Incheon 22212, Republic of Korea Department of Biological Sciences and Bioengineering, Inha University Incheon 22212 Republic of Korea; 2 Division of Biomedical Research, Korea Research Institute of Bioscience and Biotechnology, Daejeon 34141, Republic of Korea Division of Biomedical Research, Korea Research Institute of Bioscience and Biotechnology Daejeon 34141 Republic of Korea; 3 Museo Nacional de Ciencias Naturales (CSIC), Dpto. Biodiversidad y Biología Evolutiva, Madrid 28006, Spain Museo Nacional de Ciencias Naturales (CSIC), Dpto. Biodiversidad y Biología Evolutiva Madrid 28006 Spain; 4 Nakdonggang National Institute of Biological Resources, Sangju 37242, Republic of Korea Nakdonggang National Institute of Biological Resources Sangju 37242 Republic of Korea

**Keywords:** Asia, neglected habitat, subterranean biodiversity, Syncarida, taxonomy

## Abstract

**Background:**

We report the first finding of Bathynellacea, discovered in Mongolia. We also report a new species of the genus *Altainella* Camacho, 2020, which was previously recorded only in the western edge of Russia.

**New information:**

*Altainellamongoliensis* sp. nov. was found in the interstitial hyporheic region of the Onon River Basin, Mongolia, by inserting a core approximately 1.2 m deep and extracting the underground interstitial water. The new species exhibited sexual dimorphism in thoracopod VII, uniquely observed within the genus *Altainella*. We provide a morphological description and remarks on the new species with molecular information based on the 18S rDNA and partial CO1 gene sequences. We emphasise the need for continued research on the subterranean biodiversity in previously neglected regions by reporting the first discovery of macro-stygobionts in Mongolia.

## Introduction

A global review of groundwater faunal research indicated significant gaps in data from Asia, including Mongolia (Koch et al. 2024). This lack of data hinders the current understanding of the biodiversity patterns of subterranean ecosystems on a broader geographic scale. It has been difficult to evaluate the real diversity of this fauna for two reasons. First, a large part of the planet has not been prospected because of the inaccessibility of habitats and the lack of specialists in subterranean fauna (Culver and Pipan 2019). Second, the pace of species discovery and description is slow because of morphological homogeneity within subterranean lineages (Camacho et al. 2018, Camacho 2020). The discovery of new subterranean species in previously unexplored regions such as Mongolia is, therefore, crucial to fill knowledge gaps and provide insights into the diversity and biogeography of groundwater fauna.

The order Bathynellacea is a group of small, exclusively subterranean crustaceans that inhabit groundwater environments, such as caves, wells and interstitial spaces in alluvial sediments (Camacho and Valdecasas 2008, Guzik et al. 2008). These organisms are considered living fossils, with their origin dating back to the Carboniferous Period, approximately 350 million years ago (Schram 1984). Bathynellaceans are globally distributed, with representatives found on all continents, except Antarctica (Schminke and Noodt 1988, Camacho et al. 2021). They are characterised by a vermiform body shape, lack of eyes and pleopods, which are adaptations to their subterranean lifestyles (Serban 1972, Watling 2016). Despite their widespread occurrence, the diversity of Bathynellacea remains poorly understood because of the lack of focused research in several regions (Camacho 2020).

Bathynellacea has not been previously studied in Mongolia. We report the first Bathynellacea species discovered a summer groundwater survey in Mongolia in 2022 and describe a new species of the genus *Altainella* Camacho, 2020. The identification of the new species was confirmed through morphological analyses, CO1 distance and phylogenetic analysis based on 18S gene sequences. Our research contributes to expanding the distribution range of the genus *Altainella*, which was previously endemic to Russia and reduces the knowledge gap in terms of the diversity of the Bathynellacea group in Asia.

## Materials and methods

### Sampling and morphological observation

**Figure 1.**
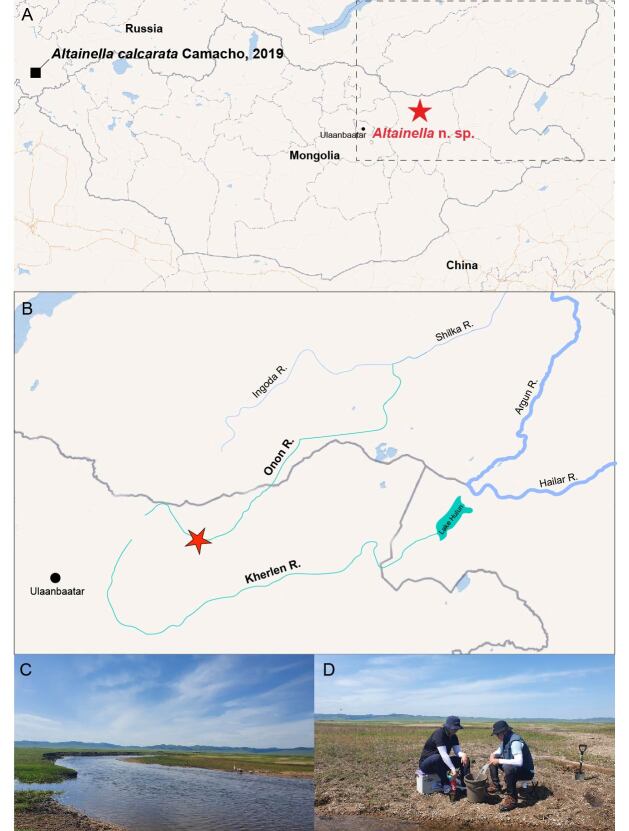
Maps and photographs of study areas. **A** Type locality of *Altainellacalcarata* (black solid square) and *A.mongoliensis* sp. nov. (red asterisk). The dotted rectangular portion is enlarged and shown in B; **B** Type locality of *Altainella* sp. nov.; **C–D** photos of the type locality of the new species.

Samples were collected from the interstitial hyporheic zone of Onon River, Mongolia (Fig. 1). The sampling and shipping of the materials were permitted by the Customs General Administration of Mongolia (MCGA). For sampling water from the hyporheic zone, a 1 m core was driven into the points using a hammer and water was collected using a manual pump and filtered using a 50 μm fine-mesh net (Lee and Park 2016). All the specimens were immediately preserved in 95% ethanol. The specimens of *Altainellamongoliensis* sp. nov. were dissected in glycerol using a stereomicroscope (SZX12, Olympus, Japan). The dissected appendages were mounted using Eukitt® Quick-hardening mounting medium (Sigma-Aldrich, St. Louis, MO, USA) for permanent slides. Observations and drawings were made using an optical microscope (DM2500; Leica, Germany). The type materials of the new species examined in this study have been deposited in collections at the Nakdonggang National Institute of Biological Resources, Korea (NNIBR) and Museo Nacional de Ciencias Naturales, Spain (MNCN).

### Molecular analysis

The abdominal tissue of each specimen was excised for the molecular study. The genomic DNA was extracted from the tissue using the LaboPass™ Tissue Genomic DNA Isolation Kit Mini (Cosmo GENETECH, Seoul, South Korea) as per the manufacturer’s instructions. Polymerase chain reaction amplification was conducted using the following primer sets: C1-J1718 and HCO2198 (Simon et al. 1994, Folmer et al. 1994) for the CO1 mitochondrial gene; 1F, 5R and 3F, 9R (Giribet et al. 1996) for the 18S nuclear gene. These sequences were aligned using ClustalW (Thompson et al. 1994, Larkin et al. 2007) in Geneious v.8.1.9 (Biomatters, Auckland, New Zealand). The uncorrected *p*-distance genetic divergence between genera was determined using MEGA X v.10.1.8 (Kumar et al. 2018). Phylogenetic analysis was performed using the Maximum Likelihood (ML) and Bayesian Inference (BI) analyses based on the 18S rRNA genes. The ML analyses were performed using ultrafast bootstrap analysis in IQ-TREE v.1.6.8, based on the TNe + R2 model. The bootstrap values were calculated with 1000 replications. Before the BI analysis, jModelTest 2.1.7 software was used to determine the appropriate DNA substitution model and the TIM2+I+G model was optimal (Darriba et al. 2012). The BI assessment was performed using MrBayes v. 3.2.6 for 1,000,000 generations (Ronquist et al. 2012); the first 30% of the generations were discarded as burn-in. The final trees are presented in FigTree v.1.4.4. and were edited using Adobe Illustrator.

## Taxon treatments

### Altainella

Camacho, 2020

BDE5D4EB-D89E-5E7F-884E-321034C2D4E5

Altainella calcarata Camacho, 2020

#### Amended diagnosis

Antennule and antenna seven segmented. Antenna equal to or longer than antennule. Mandible: mandibular palp sexually dimorphic; pars molaris formed by two small teeth near the processus incisivus accessorius and a lobe with distal region covered by denticles. Endopod of thoracopods I to VII four-segmented; coxa of thoracopod VII sexually-dimorphic, with a spur-shaped structure in the male. Male thoracopod VIII: penial region with two lobes, outer and inner lobes and frontal projection; basipod large, vertical, with frontal crest and one distal seta; exopod very elongated, with dorsal protrusion and setae; endopod with two setae. Female thoracopod VIII with very large epipod, longer than basipod. Uropod: sympod with more than four spines; endopod with three spines. Furcal rami with the first spine longer than the other four.

### Altainella mongoliensis

Ji, Camacho, Lee and Min sp. nov.

12B52C78-4B0A-5FB2-B1D1-7DB81007BDA4

3C6B12AE-FBC0-4497-A9C5-E323D9D4CE09

#### Materials

- **Type status:** Holotype. **Occurrence:** catalogNumber: Not yet; recordedBy: Ji, Camacho, Lee and Min; individualCount: 1; sex: male; lifeStage: adult; occurrenceID: 9FEAE306-3418-59AD-8419-CBB7CD56BF83; **Taxon:** scientificName: Altainellamongoliensis; phylum: Arthropoda; class: Malacostraca; order: Bathynellacea; family: Bathynellidae; genus: Altainella; specificEpithet: mongoliensis; **Location:** country: Mongolia; stateProvince: Khentii; verbatimLatitude: 48.290972; verbatimLongitude: 110.210286; **Identification:** identifiedBy: Su-Jung Ji; **Event:** eventDate: 22-07-2022; **Record Level:** language: en; institutionCode: MNCN 20.04/21011; basisOfRecord: PreservedSpecimen
- **Type status:** Holotype. **Occurrence:** catalogNumber: Not yet; recordedBy: Ji, Camacho, Lee and Min; individualCount: 1; sex: female; lifeStage: adult; occurrenceID: 09A234BD-A807-5637-8F13-8FE354D29F9C; **Taxon:** scientificName: Altainellamongoliensis; phylum: Arthropoda; class: Malacostraca; order: Bathynellacea; family: Bathynellidae; genus: Altainella; specificEpithet: mongoliensis; **Location:** country: Mongolia; stateProvince: Khentii; verbatimLatitude: 48.290972; verbatimLongitude: 110.210286; **Identification:** identifiedBy: Su-Jung Ji; **Event:** eventDate: 22-07-2022; **Record Level:** type: Paratype; language: en; institutionCode: NNIBRIV118588; basisOfRecord: PreservedSpecimen
- **Type status:** Paratype. **Occurrence:** catalogNumber: Not yet; recordedBy: Ji, Camacho, Lee and Min; individualCount: 1; sex: male; lifeStage: adult; occurrenceID: 3742219F-4318-5F5E-B2B0-AA1AA218F6DB; **Taxon:** scientificName: Altainellamongoliensis; phylum: Arthropoda; class: Malacostraca; order: Bathynellacea; family: Bathynellidae; genus: Altainella; specificEpithet: mongoliensis; **Location:** country: Mongolia; stateProvince: Khentii; verbatimLatitude: 48.290972; verbatimLongitude: 110.210286; **Identification:** identifiedBy: Su-Jung Ji; **Event:** eventDate: 22-07-2022; **Record Level:** language: en; institutionCode: MNCN 20.04/21012; basisOfRecord: PreservedSpecimen
- **Type status:** Paratype. **Occurrence:** catalogNumber: Not yet; recordedBy: Ji, Camacho, Lee and Min; individualCount: 1; sex: female; lifeStage: adult; occurrenceID: 34CF4994-0EA5-5D6A-8A7E-F2C450F4EB5C; **Taxon:** scientificName: Altainellamongoliensis; phylum: Arthropoda; class: Malacostraca; order: Bathynellacea; family: Bathynellidae; genus: Altainella; specificEpithet: mongoliensis; **Location:** country: Mongolia; stateProvince: Khentii; verbatimLatitude: 48.290972; verbatimLongitude: 110.210286; **Identification:** identifiedBy: Su-Jung Ji; **Event:** eventDate: 22-07-2022; **Record Level:** language: en; institutionCode: NNIBRIV118589; basisOfRecord: PreservedSpecimen

#### Description

##### Description of adult male

**Figure 2.**
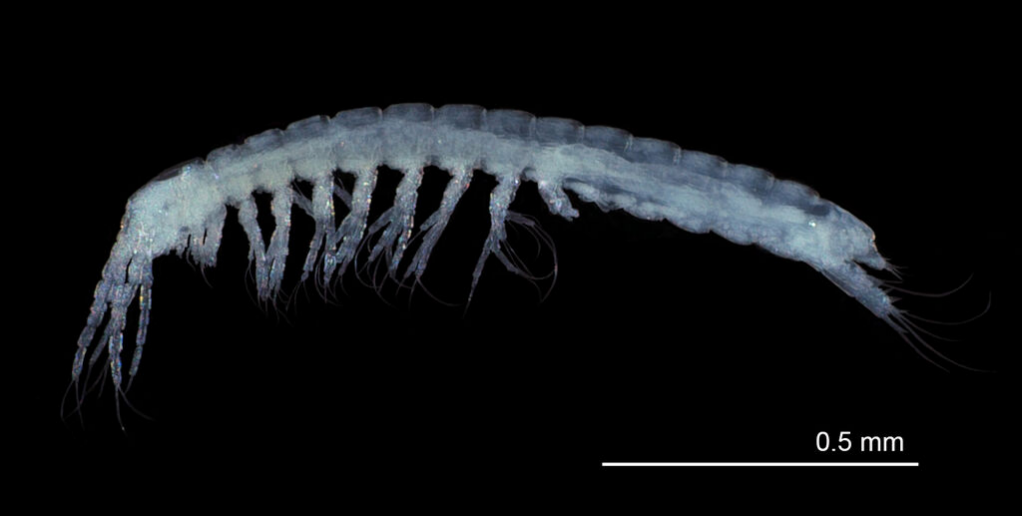
Habitus of *Altainellamongoliensis* sp. nov. (holotype).

**Total body** length 1.6 mm (Fig. 2). Body elongated, segments widening slightly towards the posterior end. Head longer than wide. Pleotelson with one small barbed dorsal seta on each side.

**Figure 3.**
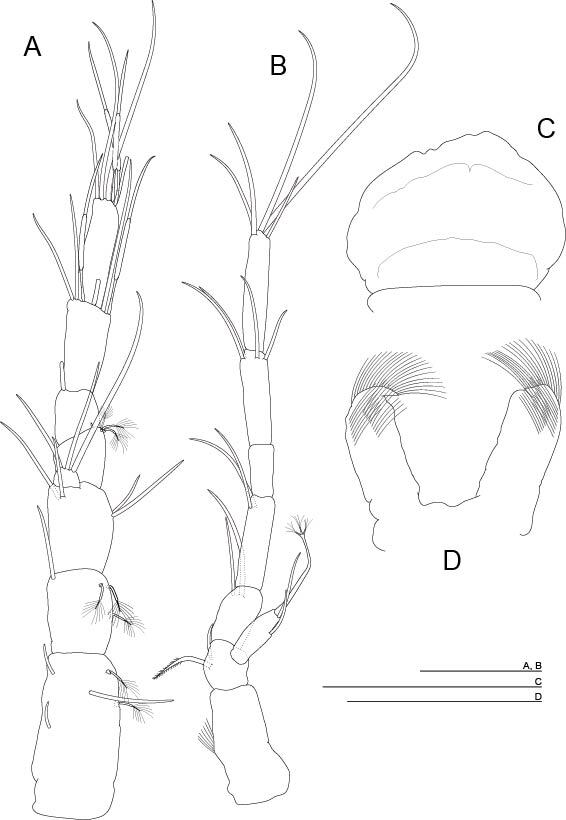
*Altainellamongoliensis* sp. nov., holotype male. **A** Antennule; **B** Antenna; **C** Labrum; **D** Paragnath. Scale bars: 0.05 mm.

**Antennule** (Fig. 3A). Seven-segmented; first three articles longer than the last four combined; first article longer than the last, which is more slender than the rest of the articles; fourth and fifth articles shortest and equal in length; small trapezoidal inner flagellum; setation as in Fig. 3A; segment three with four smooth setae; segment six with three aesthetascs, similar in size; seventh segment with three aesthetascs, similar in size. Antennule as the antenna in length.

**Antenna** (Fig. 3B). Seven-segmented; with medial seta on exopod; first article long; second and third shortest; fourth and sixth similar in length to the first; fifth small, measuring just over half the length of the distal article, which is the longest; setal formula: 0+0/2+0/2+0/2+0/0+0/2+2/5.

**Labrum** (Fig. 3C). Distal smooth free edge with irregular central protuberances.

**Paragnath** (Fig. 3D). Almost rectangular, globose, with a distal claw; setulation on the distal half.

**Figure 4.**
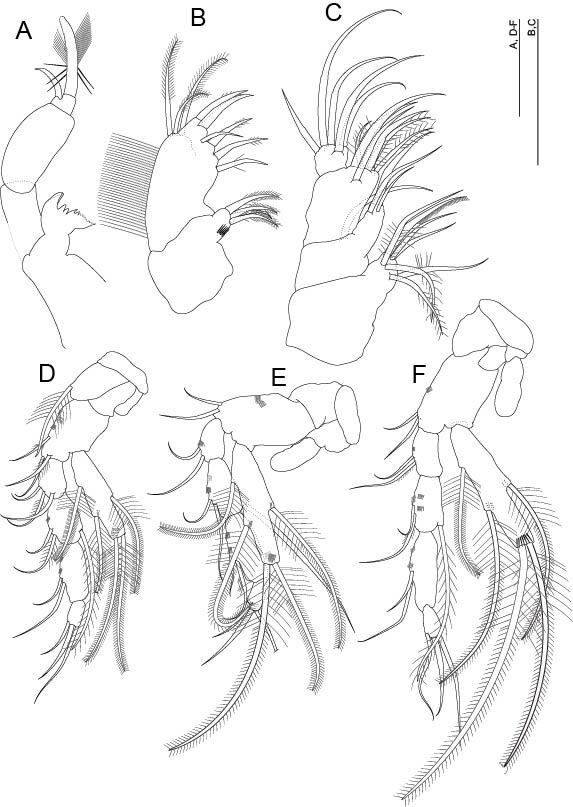
*Altainellamongoliensis* sp. nov., holotype male. **A** Mandible; **B** Maxillule; **C** Maxilla; **D** Thoracopod I; **E** Thoracopod II; **F** Thoracopod III. Scale bars: 0.05 mm.

**Mandible** (Fig. 4A). Palp with three articles, third article with two claws of different lengths, one strong and barbed, one small and smooth first and second articles rectangular and robust and third article small and almost square. Masticatory part: incisor process (*pars incisiva*) with two teeth, *processus incisivus accessorius* with two teeth, *pars molaris* with two teeth and a lobe with small denticles on the distal part.

**Maxillule** (Fig. 4B). Proximal endite with four setae, all setulose; distal endite with six teeth, four denticles; three plumose setae of similar length on the outer margin.

**Maxilla** (Fig. 4C). Four-segmented; setal formula 7, 4, 7 and 5.

**Figure 5.**
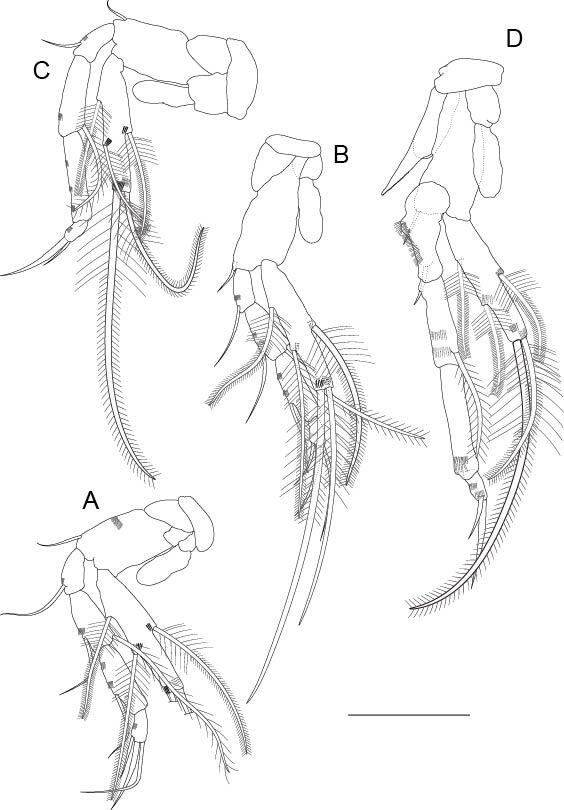
*Altainellamongoliensis* sp. nov., holotype male. **A** Thoracopod IV; **B** Thoracopod V; **C** Thoracopod VI; **D** Thoracopod VII. Scale bars: 0.05 mm.

**Thoracopods I to VII** (Fig. 4D–F and Fig. 5A–D). Well-developed; thoracopod I–III (Fig. 4D–F) progressively longer; thoracopod IV–VI (Fig. 5A–C) of similar length; thoracopod VII (Fig. 5D) slightly longer than rest and dimorphic; thoracopod I without epipod; coxa with long strong plumose seta; rectangular basipod with three smooth setae on thoracopod I, with two setae on thoracopods II and III, only one seta on thoracopods IV to VI and without seta on thoracopod VII; thoracopods II to VII with epipod, a little longer than half the basipod. Exopod of thoracopods I to VII one segmented and shorter than endopod, reaching the middle of the third endopodal article in thoracopods I to VI and as long as the first two segments combined in thoracopod VII, with five barbed setae (two terminal, one dorsal and two ventral); endopod four segmented in all thoracopods, with first three articles similar in length in thoracopod I (Fig. 4D) and second article longer than the first one in thoracopods II to V (Fig. 4E, F and Fig. 5A and B) and of equal length to the third article in thoracopods VI and VII, which is longer than the second article in thoracopods I to V; four articles very small in all thoracopods; thoracopod VII with a coxal structure like a very pointed ‘spur’ (Fig. 5D), with distal internal edge serrated, extending beyond the distal end of the basipod; first article of the endopod modified with a basal dilatation, with a seta and an external row of spines that extends to the distal end of the article. Setal formula for endopods:

Thoracopod I: (3) 3+0/2+1/2+0/3

Thoracopod II–III: (2) 2+0/2+1/2+0/3

Thoracopod IV–V: (1) 1+0/1+1/1+0/3

Thoracopod VI: (1) 1+0/0+1/0+0/2(1)

Thoracopod VII: (1) 1+0/0+1/0+0/2(1)

**Figure 6.**
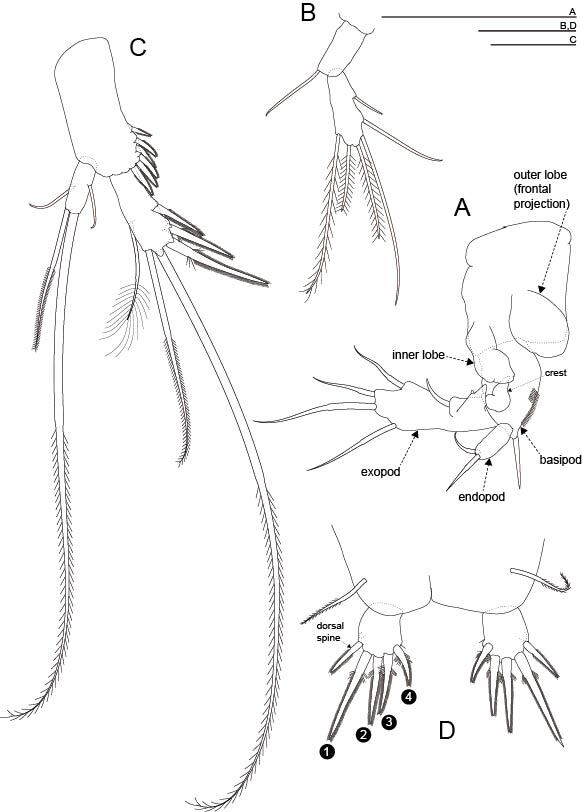
*Altainellamongoliensis* sp. nov., (A) Paratype male, (B–D) Holotype male. **A** Thoracopod VIII; **B** Pleopod; **C** Uropod; **D** Pleotelson and furcal rami. Scale bars: 0.05 mm.

**Male thoracopod VIII** (Fig. 6A). Large and long; basal region of the penial complex well-developed; a penial region with two lobes of similar length, outer and inner lobes, with the distal end of frontal projection rounded and vertical large basipod, with very small frontal crest, one distal seta at the base of the exopod and a row of small spines on inner surface from the middle of the basipod to almost the distal end; exopod very elongated, three times longer than the endopod, with five setae, two distal, two subdistal and one on a dorsal protrusion on the proximal half; endopod small, rectangular, bearing two setae.

**Pleopod** (Fig. 6B). Two segmented; the first segment with very long smooth seta; the second segment with six setae — three smooth and three barbed of different length.

**Uropod** (Fig. 6C). Sympod 60% longer than wide and 40% longer than endopod, with six equal distal spines; the endopod is almost 1.5 times longer than the exopod, with three strong spines, the distal one longer than second which is longer than the first, two distal barbed setae, the internal more than twice as long as the external one and one plumose seta located dorso-laterally; exopod with four setae, two terminals, one of them very long and two medial setae.

**Pleotelson** (Fig. 6D). One barbed dorsal seta on each side near the base of the furca, slightly longer than that of the furca.

**Furcal rami** (Fig. 6D). Almost square, bearing five spines, the first slightly longer than the second and third, which are similar in length and almost twice as long as the fourth, similar in length to the dorsal spine.

##### Description of adult female

Females have the same morphological characteristics as males, excluding the parts of the body where sexual dimorphism is manifested — the mandible and thoracopods VII and VIII.

**Figure 7.**
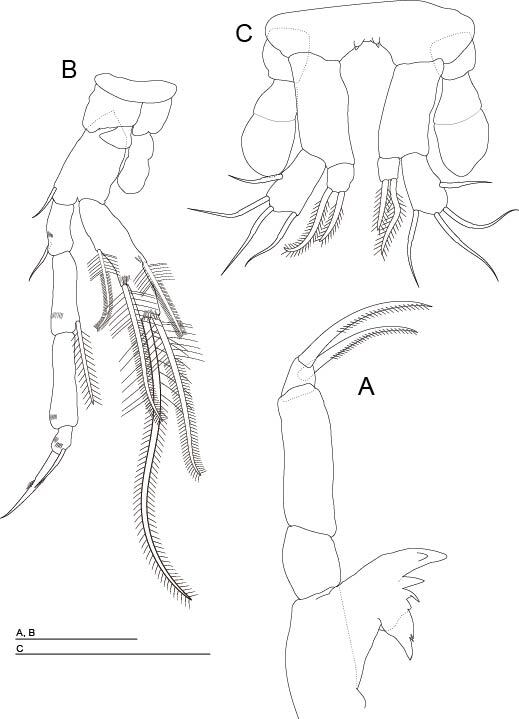
*Altainellamongoliensis* sp. nov., paratype female (NNIBRIV118589). **A** Mandible; **B** Thoracopod VII; **C** Thoracopod VIII. Scale bars: 0.05 mm.

**Mandible** (Fig. 7A) is similar to that of males, except for the second article of the mandibular palp, which is longer than that of males and bears two distal barbed claws of similar sizes.

**Thoracopod VII** (Fig. 7B) without coxal structure like ‘spur’ and with the first article of endopod unmodified.

**Thoracopod VIII** (Fig. 7C). Coxa with small tooth; very large epipod, exceeding the length of basipod; small trapezoidal endopod one-segmented with two unequal barbed apical setae; exopod 2.5 times as long as endopod and third of the length of the basipod, with three smooth apical setae similar in length and slightly shorter medial setae.

#### Diagnosis

Antennule and antenna seven segmented. Antenna exopod with median seta. Mandible: mandibular palp sexually dimorphic; pars molaris formed by two small teeth near the processus incisivus accessorius and a lobe with a distal region covered by denticles. Endopod of thoracopods I–VII four-segmented; coxapod of thoracopod VII sexually dimorphic, with a spur-shaped structure in males. Male thoracopod VIII: a penial region with two lobes of similar length, outer and inner lobes and frontal projection; basipod large, vertical, with frontal crest and one distal seta; exopod very elongated, with dorsal protrusion and setae; endopod with two setae. Female thoracopod VIII: a very large epipod, as long as an exopod, much longer than an endopod. Uropod: sympod with six spines; endopod with three spines. Furcal rami with the first spine longer than the other four.

#### Etymology

The species name "*mongoliensis*" is a combination of "Mongolia", the country from where the new species was collected and the suffix "-ensis."

#### Taxon discussion

The differences and similarities between the new species and *Altainellacalcarata* Camacho, 2020 are summarised in Table 1. Here, we review the morphological characteristics of the two species of the genus *Altainella*.

*Size and mouthparts*: Both species were similar in size and large for the average species of the family Bathynellidae. In the antennule, the first article of the new species is rectangular, whereas *A.calcarata* is small and almost square. Comparing the lengths of the antennule and antenna, *A.calcarata* had a slightly longer antenna than the antennule, whereas, they were almost similar in length in the new species. In the new species, the exopod of the antenna did not reach the third segment of the antenna; however, in *A.calcarata*, it extended well beyond the third segment. The paragnaths were thicker in the new species than in *A.calcarata*. In the new species, the male mandibular palp had a longer first article and shorter external setae in the third article compared to *A.calcarata*. The maxillule of the two *Altainella* species had four setae in the proximal segment. However, unlike *A.calcarata*, the new species has setae in the rows on the outer edge of the distal segment. The maxillae were similar in articles and setation for both species.

*Thoracopods I to VII*: Thoracopods are similar in both species, but unlike *A.calcarata*, the new species lacks an epipod on thoracopod I. The new species has reduced seta formation on the thoracopods compared to *A.calcarata*. The spur-like protrusion of the male thoracopod VII is longer and more pointed in the new species than in *A.calcarata*. The first segment of the endopod of the male thoracopod VII was highly enlarged in the new species compared to *A.calcarata*.

*Thoracopod VIII*: The exopod of female thoracopod VIII in the new species is much shorter and stubbier and the exopod is longer than that of other species. The male thoracopod VIII differs in both species, especially in the penial region, where the frontal projection of the new species is larger than that of A.calcarata, which has a smaller outer lobe and larger basipod crest.

*Pleopod*: The new species bears six setae on the second segment in pleopod, while *A.calcarata* had 12 setae.

*Furcal rami and uropod*: In the new species, the four spines on the furcal rami, excluding the dorsal spine, gradually increase in length towards the outer edge. However, in *A.calcarata*, the second and third spines are almost equal in size, whereas the first spine is nearly twice as long. In the uropod of the new species, there was only one median seta on the outer edge of both the endopod and the exopod. However, in *A.calcarata*, there were two setae each — one at the one-third and two-thirds points. This is consistent with the reduced thoracopod seta formula in the new species compared with that in the existing species.

#### Molecular data

**Figure 8.**
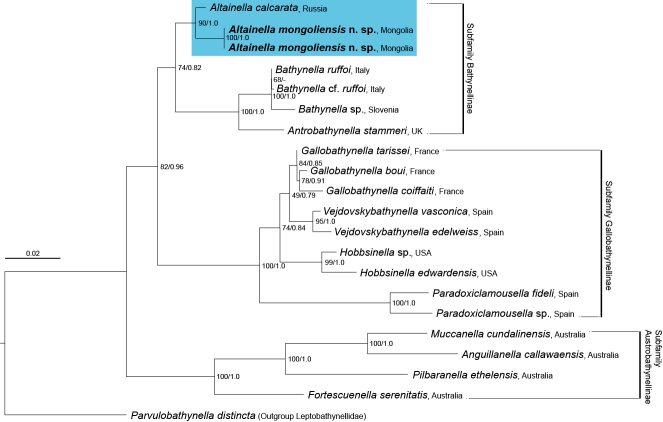
Maximum Likelihood and Bayesian Inference analyses, based on nuclear 18S gene sequences (majority consensus tree of ML). Numbers on nodes represent ultrafast bootstrap values from 1,000 replications for Maximum Likelihood and Bayesian posterior probabilities.

In this study, a single 486 bp of CO1 and two 1,701 bp of 18S gene sequences were obtained. The interspecific genetic distances between the new species and the Russian *Altainella* species were 15.0% for CO1 and 1.4% for 18S gene sequences. The two obtained 18S rRNA gene sequences were included in the Maximum Likelihood (ML) phylogenetic analysis depicted in Fig. 8. We submitted three sequences to the NCBI (National Center for Biotechnology Information) database: PQ032499 for CO1; PQ037633 and PQ037634 for 18S rDNA sequences.

#### 18S-based Maximum Likelihood (ML) analysis

The 18S rDNA alignment dataset comprised 1,433 bp derived from 21 sequences (Fig. 8, Table 2). The analysis revealed that the new species, *Altainellamongoliensis* sp. nov., formed a well-supported clade with *A.calcarata* from Russia, which was the only other known species within the genus (PP = 90). This close phylogenetic relationship between the two species suggests a strong evolutionary affinity and supports the placement of the new species. Additionally, the results of the ML analysis supported three distinct clades representing three subfamilies— Bathynellinae, Gallobathynellinae and Austrobathynellinae — within the family Bathynellidae (PP = 74, 100 and 100, respectively), which supports their morphological classification and previous molecular phylogenetic studies (Camacho et al. 2013, Camacho et al. 2020).

## Discussion

Morphological comparisons between *Altainellamongoliensis* sp. nov. and *A.calcarata* Camacho, 2020 revealed a combination of shared ancestral characters and distinct features that support their recognition as separate species within the genus. The morphological similarities between these two species, such as their large size for the Bathynellidae family and the shared structure of the antenna, paragnath, mandible and particularly the unique spur-like appendage added, based on the male thoracopod VII, supported a close evolutionary relationship between them. However, the notable differences in the maxillule and pleopod, as well as the morphology of the female and male thoracopod VIII and furcal rami, justify the recognition of *A.mongoliensis* sp. nov. as a distinct species (see Taxon discussion and Table 1).

The occurrence of these two *Altainella* species in Russia and Mongolia is intriguing from the perspective of their dispersal ability and biogeography. Despite the limited dispersal capabilities associated with subterranean habitats, the presence of these closely-related species in geographically distant locations suggests that *Altainella* may have a wider distribution than previously believed. The hydrogeological connections associated with various geological events following the Mongol-Okhotsk collision between Mongolian and Russian landmasses in the late Mesozoic era might have contributed to shaping the current distribution of the new species and *A.calcarata* (Zorin 1999, Fritzell et al. 2016).

The Mongolian region holds significant importance in the study of biodiversity and evolutionary relationships because of its unique geographic position between East Asia and Europe, providing opportunities for comparative evolutionary studies with adjacent regions. Research on Mongolian subterranean fauna is notably crucial for understanding the diversity, evolution and biogeography of the understudied Bathynellacea group across Asia. Future studies should focus on exploring additional groundwater habitats in Asia to better understand the distribution and diversity of *Altainella* and other bathynellid taxa. Integrating detailed morphological analyses with molecular data will provide a more comprehensive understanding of the evolutionary origins and biogeographic patterns of the Bathynellacea group.

## Supplementary Material

XML Treatment for Altainella

XML Treatment for Altainella mongoliensis

## Figures and Tables

**Table 1.** Differences between the two *Altainella* species.

		***Altainellacalcarata***	***Altainellamongoliensis* sp. nov.**
**Antennule**	articles number	7	7
	Aesthetacs on articles 6/7	2/2	2/3
	Setae on article 3	5	4
**Antenna**	Articles number	7	7
	Exopod: medial seta	Present	Present
	Setal formula	0+0/2+0/2+0/2+0/0+0/2+2/5	0+0/2+0/2+0/2+0/0+0/2+2/5
**Antennule vs. Antennae**		AI < AII	AI = AII
**Mandible**	Palp	3 articles	3 articles
	Teeth	5+small lobe with denticles	6+small lobe with denticles
	Sexual dimorphism	Yes, Md palp and ThVII	Yes, Md palp and ThVII
	First and third articles of palp	Square and square	Rectangular and square
**Paragnath**		Setules & tooth	Setules & tooth
**Maxillule**	Setules on the outer margin	Absent	Present
**Maxilla**	MxII: setal formula	7, 4, 7, 5	7, 4, 7, 5
**Thoracopod I - VII**	ThI–ThVII: endopod	4 articles	4 articles
	ThI: (setae basipod) setae art endp	(3) 4+0/4+1/4+0/3	(3) 3+0/2+1/2+0/3
	ThII: (setae basipod) setae art endp	(2) 3+0/3+1/3+0/3	(2) 2+0/2+1/2+0/3
	ThIII: (setae basipod) setae art endp	(2) 3+0/3+1/3+0/3	(2) 2+0/2+1/2+0/3
	ThIV: (setae basipod) setae art endp	(2) 2+0/2+1/3+0/3	(1) 1+0/1+1/1+0/3
	ThV: (setae basipod) setae art endp	(1) 2+0/2+1/2+0/3	(1) 1+0/1+1/1+0/3
	ThVI: (setae basipod) setae art endp	(1) 1+0/0+1/0+0/2(1)	(1) 1+0/0+1/0+0/2(1)
	ThVII: (setae basipod) setae art endp	(1) 0+0/0+1/0+0/2(1)	(1) 1+0/0+1/0+0/2(1)
**Thoracopod VIII female**	Female Th VIII: coxal seta	Absent	Present
	Endopod	One very long article	One almost square article
	Exopod	Very short	Short
	Setae	4	4
	Epipod	Very long	Long
**Thoracopod VIII male**	Frontal projection	Small and bilobed distal end	Very large, rounded distal end
	Basipod: crest	Large	Small
	Endopod	Small and one segment	Small and one segment
	Exopod	Like exopod of Thoracopods	Like exopod of Thoracopods
**Pleopod**	Setae in distal segment	12	6
**Uropod**	Sympod	40% longer than wide	60% longer than wide
	Sympod/endopod	Sympod = endopod	Sympod > endopod
	Spines	5	6
	Endopod	100% longer than exopod	60% longer than exopod
	Spines	3	3
	Setae number	4	3
	Exopod: setae number	5	4
**Furcal rami**	Furca: ratio first/second spines	First two times longer than second	First slightly longer than second
	Dorsal spines	Similar to the second and third	Similar to the first
	Dorsal seta of pleotelson	Longer than the furcal rami	As long as furcal rami
**Habitus**	Maximum length	1.5	1.6

**Table 2.** Data used for ML analysis.

**Species**	**Country**	**GenBank Accession number**	**Reference**
**Family Bathynellidae Grobben, 1905**			
*Altainellamongoliensis* Ji, Camacho, Lee & Min, 2024 sp. nov.	Mongolia	PQ037633, PQ037634	This study
*Altainellacalcarata* Camacho, 2020	Russia	MN262081	Camacho et al. (2020)
*Antrobathynellastammeri* (Jakobi, 1954)	UK	MF094714	Camacho et al. (2018)
Bathynellacf.ruffoi Serban, 1973	Italy	MF436213	Camacho et al. (2020)
*Bathynellaruffoi* Serban, 1973	Italy	MF436212	Camacho et al. (2020)
*Bathynella* sp.	Slovenia	MF094715	Camacho et al. (2018)
*Gallobathynellatarissei* Serban, Coineau & Delamare Deboutteville, 1971	France	KP999752	Camacho et al. (2018)
*Gallobathynellaboui* Serban, Coineau & Delamare Deboutteville, 1971	France	KP999757	Camacho et al. (2018)
*Gallobathynellacoiffaiti* Serban, Coineau & Delamare Deboutteville, 1971	France	KP999760	Camacho et al. (2018)
*Vejdovskybathynellavasconia* Camacho, Dorda & Rey, 2013	Spain	KC469516	Camacho et al. (2013)
*Vejdovskybathynellaedelweiss* Camacho, 2007	Spain	KC469512	Camacho et al. (2013)
*Paradoxiclamousellafideli* Camacho, Dorda & Rey, 2013	Spain	KC469524	Camacho et al. (2018)
*Paradoxiclamousella* sp.	Spain	JX121235	Camacho et al. (2013)
*Hobbsinella* sp. 1	USA	MN262077	Camacho et al. (2020)
*Hobbsinellaedwardensis* Camacho & Hutchins 2018	USA	KP999685	Camacho et al. (2018)
*Fortescuenellaserenitatis* Perina & Camacho, 2019	Australia	MK134941	Perina et al. (2019)
*Pilbaranellaethelensis* Perina & Camacho, 2018	Australia	MN149123	Perina et al. (2019)
*Anguillanellacallawaensis* Perina & Camacho, 2019	Australia	MF042209	Perina et al. (2019)
*Muccanellacundalinensis* Perina & Camacho, 2019	Australia	MN149112	Perina et al. (2019)
**Family Leptobathynellidae Noodt, 1965 (Outgroup)**			
*Parvulobathynelladistincta* Reddy, Bandari & Totakura, 2011	India	MF436218	Camacho et al. (2021a)
